# Low methodological quality of systematic reviews on acupuncture: a cross-sectional study

**DOI:** 10.1186/s12874-021-01437-0

**Published:** 2021-10-30

**Authors:** Leonard Ho, Fiona Y. T. Ke, Charlene H. L. Wong, Irene X. Y. Wu, Andy K. L. Cheung, Chen Mao, Vincent C. H. Chung

**Affiliations:** 1 School of Chinese Medicine, Faculty of Medicine, The Chinese University of Hong Kong, Shatin, Hong Kong; 2 The Jockey Club School of Public Health and Primary Care, Faculty of Medicine, The Chinese University of Hong Kong, Shatin, Hong Kong; 3 Xiangya School of Public Health, Central South University, 5/F, 238 Shang-Ma-Yuan-Ling Alley, Kai-Fu District, Changsha, Hunan China; 4 Department of Epidemiology, School of Public Health, Southern Medical University, Guangzhou, China

**Keywords:** Evidence-based practice, Meta-analysis, Acupuncture, Research design, Systematic reviews

## Abstract

**Background:**

While well-conducted systematic reviews (SRs) can provide the best evidence on the potential effectiveness of acupuncture, limitations on the methodological rigour of SRs may impact the trustworthiness of their conclusions. This cross-sectional study aimed to evaluate the methodological quality of a representative sample of SRs on acupuncture effectiveness.

**Methods:**

Cochrane Database of Systematic Reviews, MEDLINE, and EMBASE were searched for SRs focusing on the treatment effect of manual acupuncture or electro-acupuncture published during January 2018 and March 2020. Eligible SRs must contain at least one meta-analysis and be published in English language. Two independent reviewers extracted the bibliographical characteristics of the included SRs with a pre-designed questionnaire and appraised the methodological quality of the studies with the validated AMSTAR 2 (A MeaSurement Tool to Assess systematic Reviews 2). The associations between bibliographical characteristics and methodological quality ratings were explored using Kruskal-Wallis rank tests and Spearman’s rank correlation coefficients.

**Results:**

A total of 106 SRs were appraised. Only one (0.9%) SR was of high overall methodological quality, zero (0%) was of moderate-quality, six (5.7%) and 99 (93.4%) were of low-quality and critically low-quality respectively. Among appraised SRs, only ten (9.4%) provided an a priori protocol, four (3.8%) conducted a comprehensive literature search, five (4.7%) provided a list of excluded studies, and six (5.7%) performed meta-analysis appropriately. Cochrane SRs, updated SRs, and SRs that did not search non-English databases had relatively higher overall quality.

**Conclusions:**

Methodological quality of SRs on acupuncture is unsatisfactory. Future reviewers should improve critical methodological aspects of publishing protocols, performing comprehensive search, providing a list of excluded studies with justifications for exclusion, and conducting appropriate meta-analyses. These recommendations can be implemented via enhancing the technical competency of reviewers in SR methodology through established education approaches as well as quality gatekeeping by journal editors and reviewers. Finally, for evidence users, skills in SR critical appraisal remain to be essential as relevant evidence may not be available in pre-appraised formats.

**Supplementary Information:**

The online version contains supplementary material available at 10.1186/s12874-021-01437-0.

## Introduction

The delivery of traditional, complementary, and integrative medicine (TCIM) services in an evidence-based manner is advocated by the World Health Organization (WHO) in its *Traditional Medicine Strategy 2014–2023* [1]. As a popular form of TCIM, the use of acupuncture is increasing globally. In China, traditional Chinese medicine (TCM) constitutes a formal part of the health system, of which 20% of all outpatient services were delivered by the TCM sector, including acupuncture [2]. In Taiwan, acupuncture services are covered by the National Health Insurance [3], and the prevalence of acupuncture use was 11% in 2011 [4]. Meanwhile, in Australia [5], Germany [6], and Norway [7], acupuncture is not only regulated by the government or relevant authorities but is also partially or fully covered by statutory health insurance.

In response to the WHO’s initiative, there is a need to synthesise up-to-date evidence on the effectiveness of acupuncture, so as to facilitate the implementation of evidence-based acupuncture services. With the increasing numbers of acupuncture trials being published, keeping up with new trial results continually has become almost impossible for clinicians, managers, and policymakers [8]. Accordingly, decision-makers rely on systematic reviews (SRs) as one of the key tools for making informed decisions on the use of acupuncture interventions [9]. High-quality SRs provide a tool to assist decision-making based on a trustworthy, clear, and comprehensive synthesis of the best available evidence on a particular clinical question [9].

Although the number of SRs on acupuncture effectiveness has been increasing recently [10], there are still concerns over their methodological quality [11, 12]. For instance, inappropriate literature search, absence of critical appraisal of included primary studies, and meta-analysis of highly heterogeneous studies may give rise to biased conclusions [13, 14]. These methodological limitations may mislead decision-making in clinical practice. It is necessary to evaluate the rigour of existing SRs and consider their trustworthiness for informing decision-making.

This cross-sectional study aimed to (i) describe the bibliographical characteristics of SRs on acupuncture trials; (ii) appraise the methodological quality of SRs on acupuncture trials using AMSTAR 2 (A MeaSurement Tool to Assess systematic Reviews 2) [12].

## Methods

### Eligibility criteria

To be eligible, SRs must report at least one of the following six defining features: (i) research question; (ii) information sources searched; (iii) inclusion and exclusion criteria; (iv) screening and selection methods; (v) risk of bias assessment of the primary studies; or (vi) data synthesis and analysis methods [15]. SRs published in English with at least one meta-analysis on the treatment effect of acupuncture, including traditional manual acupuncture and electro-acupuncture, were eligible. Acupuncture refers to the use of stainless-steel filiform needles to puncture specific acupoints on the body to trigger specific therapeutic effects [16]. Meta-analysis refers to the quantitative combination of results from two or more separate trials [11]. SRs on acupuncture with moxibustion, a TCM therapy involving the burning of herbs over the skin [17], were also included. SRs on transcutaneous electrical nerve stimulation and laser acupuncture were excluded. Animal studies, narrative reviews, protocol, and network meta-analyses were also ineligible. For duplicates of SRs, the most updated versions were included for appraisal.

### Literature search

A comprehensive literature search was conducted in three international electronic databases, including the Cochrane Database of Systematic Reviews, MEDLINE, and EMBASE, for a representative sample of SRs published from January 2018 to March 2020. It is recommended that SR should be updated every two years, and hence we have chosen a sampling time frame that allowed us to focus on current SRs [11]. Details on the search strategies are shown in eTable 1, Additional file 1. The search strategies were adopted from previous SRs on acupuncture [18, 19]. Validated search filters for SRs were applied to maximise the specificity of search on MEDLINE and EMBASE [20, 21]. In this cross-sectional study of SRs, the three databases of Cochrane Database of Systematic Reviews, MEDLINE, and EMBASE were considered as the sampling frame where individual SRs were sampled. To ensure representativeness, we sampled all eligible SRs in this cross-sectional study as long as they were identified in the search using the validated search filters. This census-like sampling procedure facilitated the generation of a representative sample of SRs which are most commonly utilised by clinicians and policymakers. The use of these databases for identifying SRs is recommended in the *Comprehensive Framework of Methods for Conducting, Interpreting and Reporting Overviews* [22].

### Literature screening and data extraction

All retrieved citations were imported into Endnote X9. After deduplication, titles and abstracts of retrieved citations were screened against the eligibility criteria. Full texts of potentially eligible citations were subsequently retrieved for further assessment. For included SRs, bibliographic characteristics were extracted using a pre-designed questionnaire (eTable 2, Additional file 1) [23–26]. In academia, journal impact factor (JIF) is a widely accepted metric for measuring journal quality, evaluating the performance of researchers and institutions, and more importantly, influencing academic promotion and funding allocation [27]. Despite criticisms of its over-simplistic algorithm [28], journals with higher JIF are still regarded as more prominent in their fields [29]. Indeed, publications in journals with higher JIF are assumed to demonstrate higher methodological and reporting quality [30]. Given the existing wide acceptance of JIF, we included this as a bibliographic characteristic of SR. We also investigated the potential relationship between JIF and SR methodological quality.

Literature selection and data extraction were conducted by two independent reviewers (FYTK and AKLC). Disagreements and discrepancies were resolved via consensus between reviewers, or by arbitration of a third reviewer (CHLW).

### Methodological quality assessment

Methodological quality of included SRs was appraised by the validated AMSTAR 2 [12], across all 16 domains. AMSTAR 2 has moderate inter-rater reliability as supported by a median kappa value of 0.51 [31]. Its validity has been demonstrated by a strong positive correlation with scoring from ROBIS (A Risk of Bias Assessment Tool for Systematic Reviews) (*r* = 0.84) [31].

Seven domains were considered as critical:

i. Protocol registered before commencement of the review (item 2)
ii. Adequacy of the literature search (item 4)
iii. Justification for excluding individual studies (item 7)
iv. Risk of bias from individual studies being included in the review (item 9)
v. Appropriateness of meta-analytical methods (item 11)
vi. Consideration of risk of bias when interpreting the results of the review (item 13)
vii. Assessment of presence and likely impact of publication bias (item 15)

Based on their performance on each domain, each SRs were rated as being “high”, “moderate”, “low”, and “critically low” in terms of overall methodological quality [12], in accordance with published operational guidelines. Methodological quality assessment was conducted by two authors (FYTK and LH) independently. Disagreements and discrepancies were resolved via consensus between authors, or by arbitration of a senior researcher (VCHC).

### Data analysis

Data on bibliographical characteristics and AMSTAR 2 methodological quality assessment results were summarised using descriptive statistics. Differences in the overall methodological quality of SRs across different bibliographical characteristics were investigated using Kruskal-Wallis rank tests and Spearman’s rank correlation coefficients. A *p*-value < 0.05 was considered statistically significant. All statistical analyses were performed using SPSS 26.

## Results

### Literature selection

**Fig. 1:**
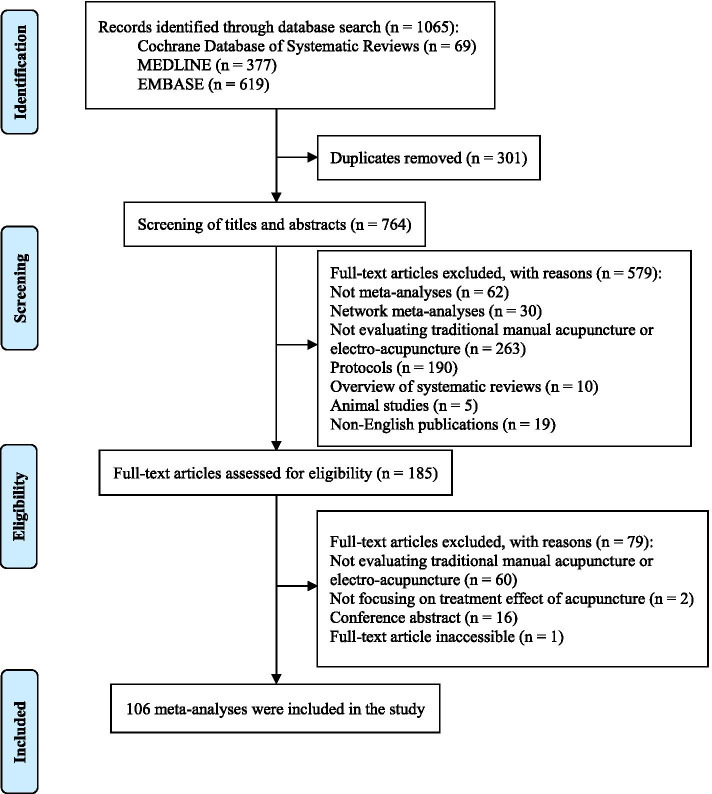
Process of literature selection

The literature search yielded a total of 1065 citations. After deduplication, titles and abstracts of 764 citations were screened. Then, 185 publications proceeded to full-text eligibility assessment. Finally, 106 SRs fulfilled the eligibility criteria and were included (eTable 3, Additional file 1). Details on literature selection are illustrated in Fig. 1. A full list of excluded records is presented in eTable 4, Additional file 1.

### Bibliographical characteristics of the included systematic reviews

The 106 included SRs contained 1864 randomised controlled trials with 204,784 participants. Only five SRs (4.7%) were Cochrane reviews. Nineteen SRs (17.9%) were an update of previous SRs. JIF ranged from 0 to 6.8 with a median of 2.0. Number of review authors ranged from two to 13 with a median of six. The corresponding authors of 93 (87.7%) SRs were from Asia, seven (6.6%) from America, four (3.8%) from Europe, and two (1.9%) from Oceania. Over a half (66; 62.3%) of the SRs had their funding sources located in Asia, while 24 (22.6%) SRs did not receive any funding support.

One-hundred-and-five (99.1%) SRs involved English database searching, while 88 (83.0%) involved non-English database searching. Most SRs reported both starting and ending years of search (81; 76.4%) and search terms for one or more electronics databases (101; 95.2%). Seventy-six (71.7%) SRs reported intervention harms. Nevertheless, 59 (55.7%) SRs did not report the language of the included primary studies.

Ninety-nine (93.4%) applied the *Cochrane risk-of-bias tool* for assessing risk of bias, two used *Jadad scale* (1.9%) or *Pedro scale* (1.9%) respectively, and two did not perform risk of bias assessment (1.9%). One-hundred-and-two (96.2%) SRs included a PRISMA (Preferred Reporting Items for Systematic Reviews and Meta-analysis) -like flow diagram to illustrate the process of literature selection. Details on bibliographical characteristics are shown in Table 1.

**Table 1:** Bibliographical characteristics of the 106 included systematic reviews on acupuncture

Bibliographical characteristics	Results^*^
**Cochrane review**	5 (4.7)
**An update of previous review**	19 (17.9)
An update of previous Cochrane review	3 (2.8)
An update of a previous non-Cochrane review	16 (15.1)
**Publication journal impact factor median (range)**	2.0 (0–6.8)
**Number of review authors median (range)**	6 (2–13)
**Location of corresponding author**	
Europe	4 (3.8)
America	7 (6.6)
Asia	93 (87.7)
Oceania	2 (1.9)
**Number of primary studies included in SRs**	
Total	1864
Median (range)	13.5 (3–73)
**Number of participants enrolled in the primary studies of SRs**	
Total	204,784
Median (range)	1238 (178–20,827)
**SRs reporting intervention harms**	76 (71.7)
**Funding location of the SR**	
Europe	4 (3.8)
America	4 (3.8)
Asia	66 (62.3)
Not reported	8 (7.5)
No funding support	24 (22.6)
**SRs that searched English databases**	105 (99.1)
**SRs that searched non-English databases**	88 (83.0)
**Report year span of search**	
Yes, reported both starting and ending years	81 (76.4)
Partially, only reported starting years	19 (17.9)
Not mentioned	6 (5.7)
**Search terms reported for one or more electronic databases**	
Topics/free text/keywords/MeSH	47 (44.3)
Full Boolean	54 (50.9)
Readers are referred elsewhere for full search strategy	0 (0)
No research term	5 (4.7)
**Language of included primary studies in SRs**	
English only	9 (8.5)
Language other than English	6 (5.7)
English and other languages	32 (30.2)
Not reported	59 (55.7)
**Risk of bias assessment tools**	
Cochrane risk of bias	99 (93.4)
Jadad scale	2 (1.9)
Pedro Scale	2 (1.9)
Others	1 (0.9)
Risk of bias assessment tool not used	2 (1.9)
**Included a PRISMA-like flow diagram**	102 (96.2)

*MeSH* Medicine Medical Subject Headings, *PRISMA* Preferred Reporting Items for Systematic Reviews and Meta-analysis, *SR* Systematic review

^*^Values are *n* (%) or median (range)

### Methodological quality

Performance among the included SRs was inadequate across four of the seven AMSTAR 2 critical domains, with that less than 20% satisfying the following: ten (9.4%) SRs established an a priori protocol and justified deviations from the protocol (item 2); four (3.8%) implemented a comprehensive literature search strategy (item 4); five (4.7%) listed excluded studies and justified the exclusions (item 7); and six (5.7%) conducted appropriate meta-analysis (item 11).

Included SRs performed relatively better across the remaining three critical domains: 97 (91.5%) had the risk of bias of individual studies assessed by appropriate instruments (item 9); 78 (73.6%) accounted for risk of bias among individual studies when interpreting results (item 13); and 23 (21.7%) investigated publication bias, and discussed its potential impact on the results (item 15).

Performance was unsatisfactory among four of the nine non-critical domains, with less than 20% fulfilling relevant criteria: four (3.8%) explained the selection of study designs for inclusion (item 3); 12 (11.3%) described included studies in adequate details (item 8); four (3.8%) reported sources of funding among individual studies included (item 10); and 19 (17.9%) assessed potential impact of risk of bias among individual studies on the results of meta-analysis (item 12).

More than 75% of SRs performed well across the remaining five non-critical domains: all SRs reported the PICO (Problem/Patient/Population, Intervention/Indicator, Comparison, and Outcome) components in their research questions and inclusion criteria (item 1); 94 (88.7%) and 97 (91.5%) SRs performed study selection (item 5) and data extraction (item 6) in duplicate, respectively; 84 (79.2%) provided a satisfactory explanation for heterogeneity in the results (item 14); and nearly all (104; 98.1%) reported the potential sources of conflict of interest (item 16). Details on the overall and individual assessment results of the included SRs are illustrated in Table 2 and Additional file 2, respectively.

**Table 2:** Results of the AMSTAR 2 items for the 106 systematic reviews on acupuncture

AMSTAR 2 items	Yes (%)	Partial Yes (%)	No (%)
1. Did the research questions and inclusion criteria for the review include the components of PICO?	106 (100)	NA	0 (0)
2. Did the report of the review contain an explicit statement that the review methods were established prior to the conduct of the review and did the report justify any significant deviations from the protocol?^a^	10 (9.4)	32 (30.2)	64 (60.4)
3. Did the review authors explain their selection of the study designs for inclusion in the review?	4 (3.8)	NA	102 (96.2)
4. Did the review authors use a comprehensive literature search strategy?^a^	4 (3.8)	99 (93.4)	3 (2.8)
5. Did the review authors perform study selection in duplicate?	94 (88.7)	NA	12 (11.3)
6. Did the review authors perform data extraction in duplicate?	97 (91.5)	NA	9 (8.5)
7. Did the review authors provide a list of excluded studies and justify the exclusions?^a^	5 (4.7)	1 (0.9)	100 (94.3)
8. Did the review authors describe the included studies in adequate detail?	12 (11.3)	84 (79.2)	10 (9.4)
9. Did the review authors use a satisfactory technique for assessing the risk of bias (RoB) in individual studies that were included in the review?^a^	97 (91.5)	3 (2.8)	6 (5.7)
10. Did the review authors report on the sources of funding for the studies included in the review?	4 (3.8)	NA	102 (96.2)
11. If meta-analysis was performed, did the review authors use appropriate methods for statistical combination of results?^a^	6 (5.7)	NA	100 (94.3)
12. If meta-analysis was performed, did the review authors assess the potential impact of RoB in individual studies on the results of the meta-analysis or other evidence synthesis?	19 (17.9)	NA	87 (82.1)
13. Did the review authors account for RoB in individual studies when interpreting / discussing the results of the review?^a^	78 (73.6)	NA	28 (26.4)
14. Did the review authors provide a satisfactory explanation for, and discussion of, any heterogeneity observed in the results of the review?	84 (79.2)	NA	22 (20.8)
15. If they performed quantitative synthesis, did the review authors carry out an adequate investigation of publication bias (small study bias) and discuss its likely impact on the results of the review?^a^	23 (21.7)	NA	83 (78.3)
16. Did the review authors report any potential sources of conflict of interest, including any funding they received for conducting the review?	104 (98.1)	NA	2 (1.9)

*AMSTAR 2* A MeaSurement Tool to Assess systematic Reviews 2, *NA* Not applicable

^a^Critical domain-specific item

### Relationship between bibliographical characteristics and overall methodological quality

Among the 106 appraised SRs, only one (0.9%) of them was of high overall methodological quality, while six (5.7%) were of low-quality. The remaining 99 (93.4%) SRs were of critically low-quality.

Results of Kruskal-Wallis tests indicated that there were statistically significant between-group differences across three bibliographical characteristics (Table 3). Cochrane reviews (*P* < 0.001), an update of a previous non-Cochrane review (*P* = 0.007), and SRs that did not search non-English databases (*P* = 0.048) had higher overall methodological quality than their counterparts. The Spearman’s rank correlation coefficient also showed that SRs published in higher JIF journals (*r*_*s*_ = 0.36; *P* < 0.001) were associated with higher overall methodological quality. No significant associations were identified between overall methodological quality and reporting of harms, funding location, year of coverage, search terms reporting, publication language restriction, risk-of-bias assessment tools used, and the inclusion of PRISMA-like flow diagram.

**Table 3:** Overall methodological quality of the 106 systematic reviews on acupuncture by bibliographical characteristics

Bibliographical characteristics	Critically low^**^**^	Low^**^**^	Moderate^**^**^	High^**^**^	***P***
**Total included SRs**	99 (93.4)	6 (5.7)	0 (0.0)	1 (0.9)	
**Cochrane Review**					< 0.001^*^
Yes	0 (0.0)	4 (80.0)	0 (0.0)	1 (20.0)	
No	99 (98.0)	2 (2.0)	0 (0.0)	0 (0)	
**An update of a previous review**					0.007^*^
Yes (Cochrane review)	3 (100)	0 (0)	0 (0.0)	0 (0.0)	
Yes (non-Cochrane review)	12 (75.0)	3 (18.8)	0 (0.0)	1 (6.3)	
No	84 (96.6)	3 (3.4)	0 (0.0)	0 (0.0)	
**Reported intervention harms**					0.659
Yes	70 (92.1)	5 (6.6)	0 (0.0)	1 (1.3)	
No	29 (96.7)	1 (3.3)	0 (0.0)	0 (0.0)	
**Funding location of the SR**					0.859
Europe	4 (100.0)	0 (0.0)	0 (0.0)	0 (0.0)	
America	4 (100.0)	0 (0.0)	0 (0.0)	0 (0.0)	
Asia	61 (92.4)	4 (6.1)	0 (0.0)	1 (1.5)	
Not reported	7 (87.5)	1 (12.5)	0 (0.0)	0 (0.0)	
No funding support	23 (95.8)	1 (4.2)	0 (0.0)	0 (0.0)	
**SRs that searched non-English databases**					0.048^*^
Yes	82 (93.2)	6 (6.8)	0 (0.0)	0 (0.0)	
No	17 (94.4)	0 (0.0)	0 (0.0)	1 (5.6)	
**Report year of coverage of literature search**					0.323
Yes	74 (91.4)	6 (7.4)	0 (0.0)	1 (1.2)	
Partially	19 (100.0)	0 (0.0)	0 (0.0)	0 (0.0)	
Not mentioned	6 (100.0)	0 (0.0)	0 (0.0)	0 (0.0)	
**Search terms reported for one or more electronic databases**					0.287
Topics/free text/keywords/MeSH	47 (100.0)	0 (0.0)	0 (0.0)	0 (0.0)	
Full Boolean	48 (88.9)	5 (9.3)	0 (0.0)	1 (1.9)	
Readers are referred elsewhere for full search strategy	0 (0.0)	0 (0.0)	0 (0.0)	0 (0.0)	
No research term	4 (80.0)	1 (20.0)	0 (0.0)	0 (0.0)	
**Eligibility criteria based on language of publication**					0.467
English only	9 (100.0)	0 (0.0)	0 (0.0)	0 (0.0)	
Language other than English	5 (83.3)	1 (16.7)	0 (0.0)	0 (0.0)	
English and other languages	28 (87.5)	3 (9.4)	0 (0.0)	1 (3.1)	
Not reported	57 (96.6)	2 (3.4)	0 (0.0)	0 (0.0)	
**Risk-of-bias assessment tools**					0.769
Cochrane risk of bias	92 (92.9)	6 (6.1)	0 (0.0)	1 (1.0)	
Jadad scale	2 (100.0)	0 (0.0)	0 (0.0)	0 (0.0)	
Pedro Scale	2 (100.0)	0 (0.0)	0 (0.0)	0 (0.0)	
Others	1 (100.0)	0 (0.0)	0 (0.0)	0 (0.0)	
Not mentioned	2 (100.0)	0 (0.0)	0 (0.0)	0 (0.0)	
**Included a PRISMA-like flow diagram**					0.865
Yes	95 (93.1)	6 (5.9)	0 (0.0)	1 (1.0)	
No	4 (100.0)	0 (0.0)	0 (0.0)	0 (0.0)	

*MeSH* National Library of Medicine Medical Subject Headings, *PRISMA* Preferred Reporting Items for Systematic Reviews and Meta-analysis, *SR* Systematic review

^^^Values are *n* (% in subgroup)

^*^P value of Kruskal-Wallis test was < 0.05

## Discussion

### Summary of results

This cross-sectional study investigated the methodological quality of a representative sample of 106 SRs on acupuncture effectiveness published between 2018 to 2020. Our results revealed that the methodological rigour of recent acupuncture SRs is weak, with more than 93% being critically low-quality. The observation that the majority of the SRs are of critically low-quality resembles findings from a similar study [32], and the floor effect caused by high standards set by the AMSTAR 2 might be an explanation. Nevertheless, such poor ratings also reflect a real need in improving SR methodology in this field [33], as only high-quality SRs should be used for guiding decision-making.

Being Cochrane review, an update of a previous non-Cochrane review, SRs that did not search non-English databases, and being published in journals with higher JIF were associated with better quality, but they only constituted a small number of SRs. In this cross-sectional study, we revealed that Cochrane reviews are likely to have a higher methodological quality than non-Cochrane reviews. It might be due to the Cochrane Collaboration’s stringent editorial requirements consisting of peer-reviewing of SR protocols [34]. This requirement acts as a gatekeeper to ensure the rigour of Cochrane reviews. Being an update of a previous SR was found to be associated with higher methodological quality as well. This observation might be attributable to improved methodological competency and experience among authors over time. The positive association between JIF and rigour echoes previous findings [30], showing the link between methodological quality and higher JIF in the context of SRs. Finally, we observed that SRs that did not search non-English databases have better rigour than their counterparts. This observation could be incidental, as it seems counterintuitive. It is known that the conduct of literature search on non-English databases is determined by the availability of funding and resources [35], and indeed these are usually more abundant in more experienced teams with more methodological expertise [11].

### Comparisons with other cross-sectional studies on systematic review rigour

The proportion of acupuncture focused SRs with high or moderate overall methodological quality (0.9%) is substantially lower than recent SRs on asthmatic treatments (15.4%) [36], osteoarthritic interventions (9.0%) [37], and osteoporosis treatments (4.0%) [26]. However, among AMSTAR 2 critical domains, SRs on acupuncture performed better than SRs of interventions for asthma, osteoporosis, and osteoarthritis [26, 36, 37]: (i) using satisfactory techniques for assessing the risk of bias in primary studies; (ii) conducting comprehensive literature search; and (iii) accounting for risk of bias among primary studies when interpreting synthesised results.

### Recommendation for future systematic reviews

#### Publishing an a priori review protocol

As SR authors tend to include primary studies with positive results [38], publication of an a priori SR protocol would reduce selective outcome reporting and enable comparison of SR protocol and its publications [11, 39]. This also minimises influence of reviewers’ biases caused by foreknowledge on preliminary results, allows peer-reviewing of planned methods, and reduces research waste due to duplication [11]. Our study showed that only 9.4% of SRs satisfied this criterion. Future authors should publish SR protocols in open-access journals, or register them on international databases [11, 12], like the International Prospective Register of Systematic Reviews (PROSPERO) [40].

#### Conducting comprehensive literature search

Only 3.8% fulfilled the criteria for completing a comprehensive literature search, as many did not conduct searches on trial registries, conference abstracts, theses, and grey literature, examining reference lists of included studies, and consulting the experts in the field of acupuncture. Such incomprehensiveness may give rise to publication bias, leading to over-estimation of effectiveness [41, 42]. On average, the exclusion of grey literature may result in an overestimation of intervention effect by approximately 12% [43]. It is also noteworthy that 55.7% did not report the language of included primary studies, which cast doubts on whether non-English publications were included. If this is the case, language bias may occur [11], leading to an overestimation or underestimation of intervention effect [44, 45]. For future acupuncture SR, ensuring a search for grey as well as non-English literature is a clear area for future improvement.

#### Providing a list of excluded studies and detailed description of included primary studies

A list of excluded studies with justifications for exclusion promotes transparency and reproducibility of SRs [46, 47]. Such a list may reduce potential publication bias and exclusion errors caused by unavoidable subjectivity during the study selection process [11, 12]. On the other hand, for included primary studies extensive details on PICO elements should be reported. These details can assist evidence users in evaluating the external validity and applicability of the findings [11], as well as in facilitating the exploration of clinical heterogeneity across primary studies [11, 12, 48]. Unfortunately, only 4.7 and 11.3% of SRs provided a list of excluded studies with rationales, or described the included primary studies in detail, respectively. Future reviewers should avoid these limitations.

#### Conducting appropriate meta-analysis

Our findings indicated that 94.3% applied inappropriate meta-analysis methods, mainly due to improper choice for a fixed-effect model. This model assumes that there is only one true effect size among all included studies, and the pooled effect estimate is common to all studies. On the contrary, a random-effect model assumes that the true effect size varies among studies, and pooled effect estimate is the mean of a distribution of true effects [49]. In the context of a meta-analysis of acupuncture trials, a random effect model is the correct choice in most of the cases. This is because some heterogeneity across trials is expected, and the assumption that there is only one true effect size is unlikely to hold [11, 49].

### Strengths and limitations

This study applied AMSTAR 2 to evaluate an up-to-date representative sample of SRs on acupuncture effectiveness. While our results indicated an urgent need to improve the methodological quality of SRs in the field, there are also several limitations. This cross-sectional study only appraised SRs published in English, and we did not search for SRs indexed in grey literature databases or prospective registers like the PROSPERO. These might have limited the representativeness of our sample. That said, it is expected that English SRs indexed in major international databases remained to be key sources of evidence impacting clinical decisions on acupuncture internationally.

In the context of SR, methodological quality and reporting quality are two distinct concepts. The former refers to the capability of an SR in providing an accurate and comprehensive summary of the results of available primary studies [12]. The latter entails the extent to which an SR described its rationale, methods, findings, and other relevant information transparently, completely, and accurately [50]. However, in this cross-sectional study, the reliability of our appraisal depended on how comprehensive the SRs reported their methodology. Poor reporting quality and journals word limit might have negatively influenced the accuracy of assessment in our sample [10].

### Implications

Most SRs on acupuncture effectiveness are of critically low methodological quality, of which these may give rise to an underestimation or overestimation of treatment effectiveness. Healthcare providers, guideline developers, and other evidence users should critically appraise the methodological quality of SRs before applying relevant evidence in policy and clinical decision-making. Journal editors and peer-reviewers are also recommended to use AMSTAR 2 and *Cochrane Handbook for Systematic Reviews of Interventions* as guidelines for verifying the quality of SRs submitted [11, 12]. Critical appraisal of SRs is time-consuming and requires specialised training which may not be available to decision-makers. To facilitate access to quality evidence, pre-appraised evidence resources have been established to inform policy or clinical decision-making. Platforms that gather appraised evidence-based information about specific clinical topics with regular updates, such as DynaMed and UpToDate, are particularly useful [51]. However, if the clinical question is yet answered by these platforms, decision-makers need to depend on SRs, and a prudent evaluation of SRs’ trustworthiness is still required.

Internationally, recommendations on the use of acupuncture have been increasing in clinical practice guidelines [52, 53]. In recent years, the Chinese Government has been upscaling the resources allocated to acupuncture research and development, as well as advocating evidence-based acupuncture practice [54]. Unfortunately, findings of this study revealed that the methodological rigour of SRs on acupuncture may not be robust enough to support these guidelines and policy initiatives. Prior to extensive implementation of evidence-based acupuncture services, clinical epidemiology education, particularly on performing high-quality clinical research and synthesis, should be emphasised. To implement the recommendations above, enhancing technical competency in SR methodology via established education approaches is warranted [11]. SR courses focusing on protocol preparation, search strategy formulation, meta-analytic methods, and result reporting have already been developed, and trainers may make use of those curriculums [55–57]. Student-led discussions, in-class activities, and student-teacher interactions are essential for increasing students’ engagement and ensuring pedagogical quality [55–57]. An evaluation of an SR methodology course showed that students were confident in selecting appropriate databases, and understanding the importance of reproducible and systematic search strategies after training, with a mean confidence score reaching 4.88 (out of 5.0) [55]. These educational outcomes were achieved with a contact time of two to three hours per week for eight to thirteen weeks [55].

## Conclusions

Methodological quality of SRs on acupuncture published in recent years is unsatisfactory, with only 0.9% of them being of high overall quality. For future SR authors, improvement efforts should focus on publishing a priori research protocols, conducting comprehensive literature search, providing lists of excluded studies with justifications for exclusion, and employing appropriate methods for meta-analysis. Technical competency of reviewers in SR methodology may be enhanced via established education approaches and quality gatekeeping by journal editors and reviewers. For evidence users, skills in SR critical appraisal remain to be essential as pre-appraised evidence may not be available.

## Supplementary Information

**Additional file 1: eTable 1.** Syntaxes for literature search. **eTable 2.** 17-item bibliographical characteristics questionnaire. **eTable 3.** List of included systematic reviews. **eTable 4.** List of excluded systematic reviews with justifications.

**Additional file 2.**

**Additional file 3:** STROBE Statement—Checklist of items that should be included in reports of ***cross-sectional studies***.

## Data Availability

The datasets used and/or analysed during the current study are available from the corresponding author on reasonable request.

## Abbreviations

AMSTAR 2: A MeaSurement Tool to Assess systematic Reviews 2
JIF: Journal impact factor
PICO: Problem/Patient/Population, Intervention/Indicator, Comparison, and Outcome
PRISMA: Preferred Reporting Items for Systematic Reviews and Meta-analysis
PROSPERO: International Prospective Register of Systematic Reviews
SR: Systematic review
ROBIS: A Risk of Bias Assessment Tool for Systematic Reviews
TCM: Traditional Chinese medicine
TCIM: Traditional, complementary, and integrative medicine
WHO: World Health Organization
