# Supplementary material for: Low methodological quality of systematic reviews on acupuncture: a cross-sectional study
Source: BMC Med Res Methodol. 2021 Oct 30;21:237. doi: 10.1186/s12874-021-01437-0 (PMC8557536; doi:10.1186/s12874-021-01437-0)
Supplement: Supplementary file 1 — Additional file 1: eTable 1. Syntaxes for literature search. eTable 2. 17-item bibliographical characteristics questionnaire. eTable 3. List of included systematic reviews. eTable 4. List of excluded systematic reviews with justifications. [file 12874_2021_1437_MOESM1_ESM.docx]

**Additional file 1**

**eTable 1.** Syntaxes for literature search

**eTable 2.** 17-item bibliographical characteristics questionnaire

**eTable 3.** List of included systematic reviews

**eTable 4.** List of excluded systematic reviews with justifications

**eTable 1.** Syntaxes for literature search

**MEDLINE**

| **#** | **Search Statement** | **Results** |
| --- | --- | --- |
| 1 | MEDLINE.tw. | 94422 |
| 2 | Systematic review.tw. | 114409 |
| 3 | meta analysis.pt. | 112011 |
| 4 | 1 or 2 or 3 | 224528 |
| 5 | exp Acupuncture/ | 1667 |
| 6 | acupunctur*.mp. | 25094 |
| 7 | exp Acupuncture Points/ | 6543 |
| 8 | exp Acupuncture Therapy/ | 24013 |
| 9 | exp Acupuncture Analgesia/ | 1201 |
| 10 | exp Electroacupuncture/ | 3871 |
| 11 | electroacupunctur*.mp. | 4636 |
| 12 | electro-acupunctur*.mp. | 727 |
| 13 | acupoint*.mp. | 4393 |
| 14 | 5 or 6 or 7 or 8 or 9 or 10 or 11 or 12 or 13 | 27424 |
| 15 | 4 and 14 | 1574 |
| 16 | limit 15 to (humans and yr="2018 -Current") | 377 |

**EMBASE**

| **#** | **Search Statement** | **Results** |
| --- | --- | --- |
| 1 | meta-analysis.tw. | 186158 |
| 2 | systematic review.tw. | 184304 |
| 3 | 1 or 2 | 296428 |
| 4 | exp Acupuncture/ | 45619 |
| 5 | acupunctur*.mp. | 45303 |
| 6 | exp Acupuncture Points/ | 1394 |
| 7 | exp Acupuncture Therapy/ | 45619 |
| 8 | exp Acupuncture Analgesia/ | 1636 |
| 9 | exp Electroacupuncture/ | 6533 |
| 10 | electroacupunctur*.mp. | 7391 |
| 11 | electro-acupunctur*.mp. | 1230 |
| 12 | acupoint*.mp. | 6353 |
| 13 | 4 or 5 or 6 or 7 or 8 or 9 or 10 or 11 or 12 | 48774 |
| 14 | 3 and 13 | 2340 |
| 15 | limit 14 to (human and yr="2018 -Current") | 619 |

**Cochrane Database of Systematic Reviews**

| **#** | **Search Statement** | **Results** |
| --- | --- | --- |
| 1 | “Acupuncture” or “acupunctur*” or “Acupuncture Points” or “Acupuncture Therapy” or “Acupuncture Analgesia” or “Electroacupuncture” or “electroacupunctur*” or “electro-acupunctur*” or “acupoint*” with Cochrane Library publication date in The last 2 years, in Cochrane Reviews | 69 |

**eTable 2.** 17-item bibliographical characteristics questionnaire

| **Bibliographical Questions** | **Answers** |
| --- | --- |
| 1. Is this a Cochrane systematic review? | Yes / No |
| 1. Is this an update of previous review? | Yes / No |
| 1. Impact factor of SR’s journal in the year before its publication | ________ / Not found / Not applicable |
| 1. Number of the SR’s authors | ________ |
| 1. Location of the corresponding author | 1. Europe 2. America 3. Asia 4. Oceania 5. Africa 6. Not reported |
| 1. Number of primary studies included in the SR | ________ |
| 1. Number of participants enrolled in the primary studies of the SR | ________ |
| 1. Has harm been considered in the SR? | Yes / No |
| 1. Funding location of the SR | 1. Europe 2. America 3. Asia 4. Oceania 5. Africa 6. Not reported |
| 1. Did the SR’s authors search for English databases? | Yes / No |
| 1. Did the SR’s authors search for non-English databases? | Yes / No |
| 1. Was “year of coverage” reported? | 1. Yes (starting and ending years) 2. Partially (only starting years) 3. Not mentioned |
| 1. Searching terms reported for one or more electronic databases | 1. No research terms 2. Topic/free text/keyword/MeSH 3. Full Boolean 4. Readers are referred elsewhere for full search strategy |
| 1. Languages of the included primary studies in the SR | 1. English only 2. Language other than English 3. English and languages other than English 4. Language criteria not reported |
| 1. Tools for assessing quality of the primary studies | 1. Cochrane RoB 2. Jaded scale 3. Schulz approach 4. Effective Public Health Practice Project Quality Assessment Tool (EPHPP) 5. Juni 6. Chalmer scale 7. Pedro Scale 8. Delphi list 9. More than one tools 10. Others |
| 1. Is there any PRISMA-like flow diagram in the SR? | Yes / No |

MeSH: Medical Subject Headings; PRISMA: Preferred Reporting Items for Systematic Reviews and Meta-Analyses; RCT: Randomised controlled trial; RoB: Risk of bias; SR: Systematic review.**eTable 3.** List of included systematic reviews

| **ID** | **Included systematic reviews** | **Availability of registered *a priori* protocol(s)** |
| --- | --- | --- |
| 1 | Acupuncture for primary insomnia: An updated systematic review of randomized controlled trials | The authors stated that an a priori review protocol was written, but not registered |
| 2 | Efficacy and safety of acupuncture for essential hypertension: A meta-analysis | The authors stated that an a priori review protocol was written, but not registered |
| 3 | Acupuncture for the treatment of sudden sensorineural hearing loss: A systematic review and meta-analysis: Acupuncture for SSNHL | *No a priori* review protocols were registered or developed |
| 4 | Effectiveness of Acupuncturing at the Sphenopalatine Ganglion Acupoint Alone for Treatment of Allergic Rhinitis: A Systematic Review and Meta-Analysis | The authors stated that an a priori review protocol was written, but not registered |
| 5 | Acupuncture and clomiphene citrate for anovulatory infertility: a systematic review and meta-analysis | *No a priori* review protocols were registered or developed |
| 6 | Acupuncture for primary trigeminal neuralgia: A systematic review and PRISMA-compliant meta-analysis | *No a priori* review protocols were registered or developed |
| 7 | Acupuncture as an adjunctive treatment for angina due to coronary artery disease: A meta-analysis | *No a priori* review protocols were registered or developed |
| 8 | Conventional treatments plus acupuncture for asthma in adults and adolescent: A systematic review and meta-analysis | *No a priori* review protocols were registered or developed |
| 9 | Optimizing acupuncture treatment for dry eye syndrome: A systematic review | *No a priori* review protocols were registered or developed |
| 10 | Acupuncture using pattern-identification for the treatment of insomnia disorder: a systematic review and meta-analysis of randomized controlled trials | *No a priori* review protocols were registered or developed |
| 11 | Efficacy and Safety of Auricular Acupuncture for Cognitive Impairment and Dementia: A Systematic Review | An *a priori* review protocol was registered on PROSPERO (CRD42017081646) |
| 12 | Acupuncture for cognitive impairment in vascular dementia, alzheimer's disease and mild cognitive impairment: A systematic review and meta-analysis | *No a priori* review protocols were registered or developed |
| 13 | Effectiveness of pharmacopuncture for cervical spondylosis: A systematic review and meta-analysis | An *a priori* review protocol was registered on PROSPERO (CRD42016043575) |
| 14 | Acupuncture combined with swallowing training for poststroke dysphagia: a meta-analysis of randomised controlled trials | *No a priori* review protocols were registered or developed |
| 15 | Scalp acupuncture treatment for children's autism spectrum disorders: A systematic review and meta-analysis | *No a priori* review protocols were registered or developed |
| 16 | Acupuncture for Post-stroke Shoulder-Hand Syndrome: A systematic review and meta-analysis | The authors stated that an a priori review protocol was written, but not registered |
| 17 | Systematic review of acupuncture for the treatment of alcohol withdrawal syndrome | The authors stated that an a priori review protocol was written, but not registered |
| 18 | Acupuncture for hip osteoarthritis | The *a priori* review protocol was a previous Cochrane Review (doi: 10.1002/14651858.CD001977.pub2.) |
| 19 | Clinical Benefits of Acupuncture for the Reduction of Hormone Therapy-Related Side Effects in Breast Cancer Patients: A Systematic Review | *No a priori* review protocols were registered or developed |
| 20 | Combination therapy of scalp electro-acupuncture and medication for the treatment of Parkinson's disease: A systematic review and meta-analysis | *No a priori* review protocols were registered or developed |
| 21 | Acupuncture for migraine: A systematic review and meta-analysis | *No a priori* review protocols were registered or developed |
| 22 | Acupuncture to Promote Recovery of Disorder of Consciousness after Traumatic Brain Injury: A Systematic Review and Meta-Analysis | An *a priori* review protocol was registered on PROSPERO (CRD42018091226) |
| 23 | Acupuncture Compared with Intramuscular Injection of Neostigmine for Postpartum Urinary Retention: A Systematic Review and Meta-Analysis of Randomized Controlled Trials | *No a priori* review protocols were registered or developed |
| 24 | Acupuncture for the relief of hot flashes in breast cancer patients: A systematic review and meta-analysis of randomized controlled trials and observational studies | *No a priori* review protocols were registered or developed |
| 25 | Effectiveness and Safety of Acupuncture for Perimenopausal Depression: A Systematic Review and Meta-Analysis of Randomized Controlled Trials | An *a priori* review protocol was registered on PROSPERO (CRD42018114506) |
| 26 | Acupuncture plus Chinese Herbal Medicine for Irritable Bowel Syndrome with Diarrhea: A Systematic Review and Meta-Analysis | *No a priori* review protocols were registered or developed |
| 27 | Electroacupuncture as an adjunctive therapy for motor dysfunction in acute stroke survivors: A systematic review and meta-analyses | The authors stated that an a priori review protocol was written, but not registered |
| 28 | Acupuncture for Acute Pancreatitis: A Systematic Review and Meta-analysis | *No a priori* review protocols were registered or developed |
| 29 | Acupuncture for chronic fatigue syndrome: a systematic review and meta-analysis | *No a priori* review protocols were registered or developed |
| 30 | Compare the efficacy of acupuncture with drugs in the treatment of Bell's palsy: A systematic review and meta-analysis of RCTs | *No a priori* review protocols were registered or developed |
| 31 | The effectiveness and safety of acupuncture for the treatment of myasthenia gravis: a systematic review and meta-analysis of randomized controlled trials | The authors stated that an a priori review protocol was written, but not registered |
| 32 | Acupuncture therapy for fibromyalgia: A systematic review and meta-analysis of randomized controlled trials | The authors stated that an a priori review protocol was written, but not registered |
| 33 | The effectiveness of acupuncture therapy in patients with post-stroke depression: An updated meta-analysis of randomized controlled trials | *No a priori* review protocols were registered or developed |
| 34 | Fire needling for herpes zoster: A systematic review and meta-analysis of randomized clinical trials | *No a priori* review protocols were registered or developed |
| 35 | Comparison between the Effects of Acupuncture Relative to Other Controls on Irritable Bowel Syndrome: A Meta-Analysis | *No a priori* review protocols were registered or developed |
| 36 | Manual Acupuncture for Optic Atrophy: A Systematic Review and Meta-Analysis | *No a priori* review protocols were registered or developed |
| 37 | Effect of warm needling therapy and acupuncture in the treatment of peripheral facial paralysis: A systematic review and meta-analysis | *No a priori* review protocols were registered or developed |
| 38 | The Effect of Acupuncture in Breast Cancer-Related Lymphoedema (BCRL): A Systematic Review and Meta-Analysis | *No a priori* review protocols were registered or developed |
| 39 | The Efficacy of Acupuncture in Chemotherapy-Induced Peripheral Neuropathy: Systematic Review and Meta-Analysis | *No a priori* review protocols were registered or developed |
| 40 | The maintenance effect of acupuncture on breast cancer-related menopause symptoms: a systematic review | *No a priori* review protocols were registered or developed |
| 41 | The effectiveness of acupuncture in the management of persistent regional myofascial head and neck pain: A systematic review and meta-analysis | The authors stated that an a priori review protocol was written, but not registered |
| 42 | Acupuncture for the Treatment of Adults with Posttraumatic Stress Disorder: A Systematic Review and Meta-Analysis | The authors stated that an a priori review protocol was written, but not registered |
| 43 | The effectiveness of superficial versus deep dry needling or acupuncture for reducing pain and disability in individuals with spine-related painful conditions: a systematic review with meta-analysis | The authors stated that an a priori review protocol was written, but not registered |
| 44 | Effects of dry needling trigger point therapy in the shoulder region on patients with upper extremity pain and dysfunction: a systematic review with meta-analysis | The authors stated that an a priori review protocol was written, but not registered |
| 45 | Is dry needling effective for low back pain?: A systematic review and PRISMA-compliant meta-analysis | *No a priori* review protocols were registered or developed |
| 46 | The effectiveness and safety of acupuncture for patients with atopic eczema: a systematic review and meta-analysis | The authors stated that an a priori review protocol was written, but not registered |
| 47 | Comparing verum and sham acupuncture in fibromyalgia syndrome: a systematic review and meta-analysis | The authors stated that an a priori review protocol was written, but not registered |
| 48 | Acupuncture for symptomatic gastroparesis | An *a priori* review protocol was registered on Cochrane Database of Systematic Reviews (doi: 10.1002/14651858.CD009676) |
| 49 | The Efficacy and Safety of Acupuncture for the Treatment of Children with Autism Spectrum Disorder: A Systematic Review and Meta-Analysis | The authors stated that an a priori review protocol was written, but not registered |
| 50 | Acupuncture Versus Sham-acupuncture: A Meta-analysis on Evidence for Non-immediate Effects of Acupuncture in Musculoskeletal Disorders | The authors stated that an a priori review protocol was written, but not registered |
| 51 | Acupuncture Treatment for Post-Stroke Dysphagia: An Update Meta-Analysis of Randomized Controlled Trials | *No a priori* review protocols were registered or developed |
| 52 | Effectiveness of Acupuncture Used for the Management of Postpartum Depression: A Systematic Review and Meta-Analysis | *No a priori* review protocols were registered or developed |
| 53 | Clinical effects and safety of electroacupuncture for the treatment of post-stroke depression: a systematic review and meta-analysis of randomised controlled trials | The authors stated that an a priori review protocol was written, but not registered |
| 54 | Placebo effect of acupuncture on insomnia: a systematic review and meta-analysis | The authors stated that an a priori review protocol was written, but not registered |
| 55 | Acupuncture for Chronic Pain-Related Insomnia: A Systematic Review and Meta-Analysis | *No a priori* review protocols were registered or developed |
| 56 | Evidence for Dry Needling in the Management of Myofascial Trigger Points Associated With Low Back Pain: A Systematic Review and Meta-Analysis | *No a priori* review protocols were registered or developed |
| 57 | Warm needle acupuncture in primary osteoporosis management: a systematic review and meta-analysis | The authors stated that an a priori review protocol was written, but not registered |
| 58 | Acupuncture for overactive bladder in adults: a systematic review and meta-analysis | *No a priori* review protocols were registered or developed |
| 59 | Traditional acupuncture for menopausal hot flashes: A systematic review and meta-analysis of randomized controlled trials | *No a priori* review protocols were registered or developed |
| 60 | The effectiveness of acupuncture for osteoporosis: A systematic review and meta-analysis | *No a priori* review protocols were registered or developed |
| 61 | Long-term effects of acupuncture for chronic prostatitis/chronic pelvic pain syndrome: Systematic review and single-Arm meta-Analyses | *No a priori* review protocols were registered or developed |
| 62 | Does acupuncture the day of embryo transfer affect the clinical pregnancy rate? Systematic review and meta-analysis | *No a priori* review protocols were registered or developed |
| 63 | Acupuncture treatments for infantile colic: a systematic review and individual patient data meta-analysis of blinding test validated randomised controlled trials | The authors stated that an a priori review protocol was written, but not registered |
| 64 | Acupuncture performed around the time of embryo transfer: a systematic review and meta-analysis | *No a priori* review protocols were registered or developed |
| 65 | Is Acupuncture Effective for Improving Insulin Resistance? A Systematic Review and Meta-analysis | *No a priori* review protocols were registered or developed |
| 66 | Efficacy of acupuncture in the management of post-apoplectic aphasia: A systematic review and meta-analysis of randomized controlled trials | *No a priori* review protocols were registered or developed |
| 67 | Acupuncture for lumbar disc herniation: a systematic review and meta-analysis | *No a priori* review protocols were registered or developed |
| 68 | Traditional Chinese acupuncture and postpartum depression: A systematic review and meta-analysis | *No a priori* review protocols were registered or developed |
| 69 | Acupuncture for Chronic Pain: Update of an Individual Patient Data Meta-Analysis | *No a priori* review protocols were registered or developed |
| 70 | Acupuncture Therapy for Functional Effects and Quality of Life in COPD Patients: A Systematic Review and Meta-Analysis | The authors stated that an a priori review protocol was written, but not registered |
| 71 | Electroacupuncture for Reflex Sympathetic Dystrophy after Stroke: A Meta-Analysis | *No a priori* review protocols were registered or developed |
| 72 | The Effect of Patient Characteristics on Acupuncture Treatment Outcomes | *No a priori* review protocols were registered or developed |
| 73 | The efficacy and safety of acupuncture in women with primary dysmenorrhea: A systematic review and meta-analysis | The authors stated that an a priori review protocol was written, but not registered |
| 74 | Role of acupuncture in the treatment of insulin resistance: A systematic review and meta-analysis | *No a priori* review protocols were registered or developed |
| 75 | Appropriateness of sham or placebo acupuncture for randomized controlled trials of acupuncture for nonspecific low back pain: A systematic review and meta-analysis | An *a priori* review protocol was registered on PROSPERO (CRD42017070122) |
| 76 | Evidence of efficacy of acupuncture in the management of low back pain: a systematic review and meta-analysis of randomised placebo- or sham-controlled trials | The authors stated that an a priori review protocol was written, but not registered |
| 77 | The effects of acupuncture on pregnancy outcomes of in vitro fertilization: A systematic review and meta-analysis | *No a priori* review protocols were registered or developed |
| 78 | Acupuncture for migraine without aura: a systematic review and meta-analysis | The authors stated that an a priori review protocol was written, but not registered |
| 79 | Acupuncture for acute stroke | An *a priori* review protocol was registered on Cochrane Database of Systematic Reviews (doi: 10.1002/14651858.CD003317) |
| 80 | Acupuncture at Tiaokou (ST38) for Shoulder Adhesive Capsulitis: What Strengths Does It Have? A Systematic Review and Meta-Analysis of Randomized Controlled Trials | *No a priori* review protocols were registered or developed |
| 81 | Acupuncture for hypertension | An *a priori* review protocol was registered on Cochrane Database of Systematic Reviews (doi: 10.1002/14651858.CD008821) |
| 82 | The effect of acupuncture on Bell's palsy: An overall and cumulative meta-analysis of randomized controlled trials | *No a priori* review protocols were registered or developed |
| 83 | Effects of acupuncture on cancer-related fatigue: a meta-analysis | *No a priori* review protocols were registered or developed |
| 84 | Acupuncture for adults with overactive bladder | The authors stated that an a priori review protocol was written, but not registered |
| 85 | Electroacupuncture for Postoperative Urinary Retention: A Systematic Review and Meta-Analysis | The authors stated that an a priori review protocol was written, but not registered |
| 86 | Meta-Analysis of Electroacupuncture in Cardiac Anesthesia and Intensive Care | *No a priori* review protocols were registered or developed |
| 87 | Acupuncture therapy improves health-related quality of life in patients with chronic obstructive pulmonary disease: A systematic review and meta-analysis | *No a priori* review protocols were registered or developed |
| 88 | The effect of acupuncture on the quality of life in patients with migraine: A systematic review and meta-analysis | *No a priori* review protocols were registered or developed |
| 89 | Cognitive improvement effects of electro-acupuncture for the treatment of MCI compared with Western medications: A systematic review and Meta-analysis 11 Medical and Health Sciences 1103 Clinical Sciences | *No a priori* review protocols were registered or developed |
| 90 | Oriental herbal medicine and moxibustion for polycystic ovary syndrome: A meta-analysis | *No a priori* review protocols were registered or developed |
| 91 | The Effect of Acupuncture and Moxibustion on Heart Function in Heart Failure Patients: A Systematic Review and Meta-Analysis | The authors stated that an a priori review protocol was written, but not registered |
| 92 | Acupuncture therapy for the treatment of stable angina pectoris: An updated meta-analysis of randomized controlled trials | *No a priori* review protocols were registered or developed |
| 93 | Traditional manual acupuncture combined with rehabilitation therapy for shoulder hand syndrome after stroke within the Chinese healthcare system: a systematic review and meta-analysis | *No a priori* review protocols were registered or developed |
| 94 | Effects of moxibustion on pain behaviors in patients with rheumatoid arthritis: A meta-analysis | *No a priori* review protocols were registered or developed |
| 95 | Acupuncture Treatment for Chronic Pelvic Pain in Women: A Systematic Review and Meta-Analysis of Randomized Controlled Trials | The authors stated that an a priori review protocol was written, but not registered |
| 96 | The effectiveness of dry needling for patients with orofacial pain associated with temporomandibular dysfunction: a systematic review and meta-analysis | The authors stated that an a priori review protocol was written, but not registered |
| 97 | Acupuncture for postherpetic neuralgia systematic review and meta-analysis | The authors stated that an a priori review protocol was written, but not registered |
| 98 | Acupoint selection for the treatment of dry eye: A systematic review and meta-analysis of randomized controlled trials | *No a priori* review protocols were registered or developed |
| 99 | Warm-needle moxibustion for spasticity after stroke: A systematic review of randomized controlled trials | *No a priori* review protocols were registered or developed |
| 100 | Acupuncture for menstrual migraine: a systematic review | *No a priori* review protocols were registered or developed |
| 101 | The efficacy of acupuncture for stable angina pectoris: A systematic review and meta-analysis | The authors stated that an a priori review protocol was written, but not registered |
| 102 | Acupuncture and weight loss in Asians: A PRISMA-compliant systematic review and meta-analysis | *No a priori* review protocols were registered or developed |
| 103 | Effects of Acupuncture on Breast Cancer-Related lymphoedema: A Systematic Review and Meta-Analysis | *No a priori* review protocols were registered or developed |
| 104 | Acupuncture for infertile women without undergoing assisted reproductive techniques (ART): A systematic review and meta-analysis | *No a priori* review protocols were registered or developed |
| 105 | Moxibustion for alleviating side effects of chemotherapy or radiotherapy in people with cancer | An *a priori* review protocol was registered on Cochrane Database of Systematic Reviews (doi: 10.1002/14651858.CD010559) |
| 106 | Acupuncture for stable angina pectoris: A systematic review and meta-analysis | The authors stated that an a priori review protocol was written, but not registered |

**eTable 4.** List of excluded systematic reviews with justifications

| **Excluded systematic reviews** | **Justifications** |
| --- | --- |
| 1. Accuracy and Precision in Acupuncture Point Location: A Critical Systematic Review | Not evaluating on traditional needle acupuncture or electro-acupuncture |
| 1. Acupoint embedding therapy improves nonalcoholic fatty liver disease with abnormal transaminase: A PRISMA-compliant systematic review and meta-analysis | Not evaluating on traditional needle acupuncture or electro-acupuncture |
| 1. Acupotomy for the treatment of lumbar spinal stenosis: A systematic review and meta-analysis | Not evaluating on traditional needle acupuncture or electro-acupuncture |
| 1. Acupotomy therapy for joint pain relief of lumbar disc herniation-systematic review and meta-analysis | Not evaluating on traditional needle acupuncture or electro-acupuncture |
| 1. Acupuncture for Acne Vulgaris: A Systematic Review and Meta-Analysis | Not evaluating on traditional needle acupuncture or electro-acupuncture |
| 1. Acupuncture for acne vulgaris: findings from a systematic review and meta-analysis | Not evaluating on traditional needle acupuncture or electro-acupuncture |
| 1. Acupuncture for anxiety in dental patients: Systematic review and meta-analysis | Not evaluating on traditional needle acupuncture or electro-acupuncture |
| 1. Acupuncture for Breathlessness in Advanced Diseases: A Systematic Review and Meta-analysis | Not evaluating on traditional needle acupuncture or electro-acupuncture |
| 1. Acupuncture for cerebral palsy: A meta-analysis of randomized controlled trials | Not evaluating on traditional needle acupuncture or electro-acupuncture |
| 1. Acupuncture for depression | Not evaluating on traditional needle acupuncture or electro-acupuncture |
| 1. Acupuncture for depression: A systematic review and meta-analysis | Not evaluating on traditional needle acupuncture or electro-acupuncture |
| 1. Acupuncture for illicit drug withdrawal syndrome: A systematic review and meta-analysis | Not evaluating on traditional needle acupuncture or electro-acupuncture |
| 1. Acupuncture for Premenstrual Syndrome at Different Intervention Time: A Systemic Review and Meta-Analysis | Not evaluating on traditional needle acupuncture or electro-acupuncture |
| 1. Acupuncture for smoking cessation: A systematic review and meta-analysis of 24 randomized controlled trials | Not evaluating on traditional needle acupuncture or electro-acupuncture |
| 1. Acupuncture for treatment of erectile dysfunction: a systematic review and meta-analysis | Not evaluating on traditional needle acupuncture or electro-acupuncture |
| 1. Acupuncture in improving endometrial receptivity: A systematic review and meta-analysis | Not evaluating on traditional needle acupuncture or electro-acupuncture |
| 1. Acupuncture in postoperative pain management-a systematic review and meta-analysis | Not evaluating on traditional needle acupuncture or electro-acupuncture |
| 1. Acupuncture on Obesity: Clinical Evidence and Possible Neuroendocrine Mechanisms | Not evaluating on traditional needle acupuncture or electro-acupuncture |
| 1. Acupuncture therapy for breast cancer-related lymphedema: A systematic review and meta-analysis | Not evaluating on traditional needle acupuncture or electro-acupuncture |
| 1. Acupuncture Therapy plus Hyaluronic Acid Injection for Knee Osteoarthritis: A Meta-Analysis of Randomized Controlled Trials | Not evaluating on traditional needle acupuncture or electro-acupuncture |
| 1. Acupuncture versus sham acupuncture for simple obesity: A systematic review and meta-analysis | Not evaluating on traditional needle acupuncture or electro-acupuncture |
| 1. The Application of Auriculotherapy to the Treatment of Chronic Spontaneous Urticaria: A Systematic Review and Meta-analysis | Not evaluating on traditional needle acupuncture or electro-acupuncture |
| 1. Auricular acupressure for myopia in children and adolescents: A systematic review | Not evaluating on traditional needle acupuncture or electro-acupuncture |
| 1. Auricular acupuncture for chronic back pain in adults: a systematic review and metanalysis | Not evaluating on traditional needle acupuncture or electro-acupuncture |
| 1. Clinical efficacy of acupoint embedment in weight control: A systematic review and meta-analysis | Not evaluating on traditional needle acupuncture or electro-acupuncture |
| 1. Clinical Evidence for Association of Acupuncture and Acupressure with Improved Cancer Pain: A Systematic Review and Meta-Analysis | Not evaluating on traditional needle acupuncture or electro-acupuncture |
| 1. Cochrane reviews on acupuncture therapy for pain: A snapshot of the current evidence | Not evaluating on traditional needle acupuncture or electro-acupuncture |
| 1. Conventional Acupuncture for Cardiac Arrhythmia: A Systematic Review of Randomized Controlled Trials | Not evaluating on traditional needle acupuncture or electro-acupuncture |
| 1. The Economic Burden of Chronic Idiopathic Constipation in the Us: A Systematic Literature Review | Not evaluating on traditional needle acupuncture or electro-acupuncture |
| 1. Effectiveness and adequacy of blinding in the moderation of pain outcomes: Systematic review and meta-analyses of dry needling trials | Not evaluating on traditional needle acupuncture or electro-acupuncture |
| 1. The Effectiveness and Safety of Acupoint Catgut Embedding for the Treatment of Postmenopausal Osteoporosis: A Systematic Review and Meta-Analysis | Not evaluating on traditional needle acupuncture or electro-acupuncture |
| 1. The Effectiveness of Acupoint Catgut Embedding Therapy for Abdominal Obesity: A Systematic Review and Meta-Analysis | Not evaluating on traditional needle acupuncture or electro-acupuncture |
| 1. Effectiveness of Acupuncture on Pain, Physical Function and Health-Related Quality of Life in Patients with Rheumatoid Arthritis: A Systematic Review of Quantitative Evidence | Not evaluating on traditional needle acupuncture or electro-acupuncture |
| 1. Effectiveness of Perioperative Auricular Therapy on Postoperative Pain after Total Hip Arthroplasty: A Systematic Review and Meta-Analysis of Randomised Controlled Trials | Not evaluating on traditional needle acupuncture or electro-acupuncture |
| 1. Effects of acupuncture on Luteinized Unruptured Follicle Syndrome: A meta-analysis of randomized controlled trials | Not evaluating on traditional needle acupuncture or electro-acupuncture |
| 1. Effects of auriculotherapy on weight and body mass index reduction in patients with overweight or obesity: Systematic review and meta-analysis | Not evaluating on traditional needle acupuncture or electro-acupuncture |
| 1. Efficacy and safety of acupuncture for the treatment of oligoasthenozoospermia: A systematic review | Not evaluating on traditional needle acupuncture or electro-acupuncture |
| 1. The efficacy of absorbable barbed suture versus traditional absorbable suture in total knee arthroplasty: A meta-analysis | Not evaluating on traditional needle acupuncture or electro-acupuncture |
| 1. Efficacy of acupotomy for cerebral palsy: A systematic review and meta-analysis | Not evaluating on traditional needle acupuncture or electro-acupuncture |
| 1. Efficacy of acupuncture for treating opioid use disorder in adults: A systematic review and meta-analysis | Not evaluating on traditional needle acupuncture or electro-acupuncture |
| 1. Electroacupuncture or transcutaneous electroacupuncture for postoperative ileus after abdominal surgery: A systematic review and meta-analysis | Not evaluating on traditional needle acupuncture or electro-acupuncture |
| 1. Invasive and non-invasive acupuncture techniques for pain management in neonates: a systematic review | Not evaluating on traditional needle acupuncture or electro-acupuncture |
| 1. Is acupuncture an effective postherpetic neuralgia treatment? A systematic review and meta-analysis | Not evaluating on traditional needle acupuncture or electro-acupuncture |
| 1. Is there a risk of transfusion-transmissible infections after percutaneous needle treatments in blood donors? A systematic review and meta-analysis | Not evaluating on traditional needle acupuncture or electro-acupuncture |
| 1. Laser Acupuncture for Patients with Knee Osteoarthritis: A Systematic Review and Meta-Analysis of Randomized Placebo-Controlled Trials | Not evaluating on traditional needle acupuncture or electro-acupuncture |
| 1. Positive effects and molecular mechanisms of traditional Chinese medicine on embryo implantation | Not evaluating on traditional needle acupuncture or electro-acupuncture |
| 1. A systematic review and meta-analysis of using acupuncture and acupressure for uremic pruritus | Not evaluating on traditional needle acupuncture or electro-acupuncture |
| 1. Systematic review of acupuncture placebo devices with a focus on the credibility of blinding of healthy participants and/or acupuncturists | Not evaluating on traditional needle acupuncture or electro-acupuncture |
| 1. Wet cupping for hypertension: a systematic review and meta-analysis | Not evaluating on traditional needle acupuncture or electro-acupuncture |
| 1. Acupuncture and moxibustion for primary osteoporosis: An overview of systematic review | Not evaluating on traditional needle acupuncture or electro-acupuncture |
| 1. Acupuncture and Related Therapies for Treatment of Postoperative Ileus in Colorectal Cancer: A Systematic Review and Meta-Analysis of Randomized Controlled Trials | Not evaluating on traditional needle acupuncture or electro-acupuncture |
| 1. Acupuncture or acupressure for pain management during labour | Not evaluating on traditional needle acupuncture or electro-acupuncture |
| 1. Acupuncture versus propranolol in migraine prophylaxis: an indirect treatment comparison meta-analysis | Not evaluating on traditional needle acupuncture or electro-acupuncture |
| 1. Clinical evidence of Chinese medicine therapies for depression in women during perimenopause and menopause | Not evaluating on traditional needle acupuncture or electro-acupuncture |
| 1. Danggui-Shaoyao-San for dementia: A PRISMA-compliant systematic review and meta-analysis | Not evaluating on traditional needle acupuncture or electro-acupuncture |
| 1. Effect of acupuncture and intervention types on weight loss: a systematic review and meta-analysis | Not evaluating on traditional needle acupuncture or electro-acupuncture |
| 1. Effectiveness and safety of ear acupuncture for trauma-related mental disorders after large-scale disasters: A PRISMA-compliant systematic review | Not evaluating on traditional needle acupuncture or electro-acupuncture |
| 1. Effectiveness of interventions for non-specific low back pain in older adults. A systematic review and meta-analysis | Not evaluating on traditional needle acupuncture or electro-acupuncture |
| 1. Filiform needle acupuncture for copd: A systematic review and meta-analysis | Not evaluating on traditional needle acupuncture or electro-acupuncture |
| 1. The Neuroscience of Nonpharmacological Traditional Chinese Therapy (NTCT) for Major Depressive Disorder: A Systematic Review and Meta-Analysis | Not evaluating on traditional needle acupuncture or electro-acupuncture |
| 1. The impact of STRICTA and CONSORT on reporting of randomised control trials of acupuncture: a systematic methodological evaluation | Not evaluating on traditional needle acupuncture or electro-acupuncture |
| 1. Meta-analysis of the effect of distal or local point selection on acupuncture efficacy | Not evaluating on traditional needle acupuncture or electro-acupuncture |
| 1. Traditional manual acupuncture combined with rehabilitation therapy for shoulder hand syndrome after stroke within the Chinese healthcare system: a systematic review and meta-analysis | Full-text article inaccessible |
| 1. Acupuncture for children with primary nocturnal enuresis: A systematic review and meta-analysis | Conference abstract |
| 1. Acupuncture treatment for chronic atrophic gastritis: A systematic review and meta-analysis of randomized controlled trials | Conference abstract |
| 1. Acupunctureand dryneedlingfor plantar heel pain: A systematic review and meta-analysis | Conference abstract |
| 1. Effect of acupuncture for urinary retention after stroke: A systematic review | Conference abstract |
| 1. The effects of acupuncture on xerostomia, xerophthalmia and antibody modification in patients with Sjogren syndrome: A metaanalysis | Conference abstract |
| 1. Management of Menopause Symptoms with Acupuncture: An Umbrella Systematic Review and Meta-Analysis | Conference abstract |
| 1. Performing a large number of overlapping meta-analyses assessing acupuncture efficacy for smoking cessation to explore vibration of effects | Conference abstract |
| 1. Role of acupuncture in acute injury management of strains and sprains | Conference abstract |
| 1. Scalp acupuncture for post-stroke cognitive impairment: A systematic review and meta-analysis | Conference abstract |
| 1. Acupuncture for chronic hepatitis B | Conference abstract |
| 1. Acupuncture for chronic pelvic inflammatory disease | Conference abstract |
| 1. Acupuncture on treating angina pectoris: A systematic review | Conference abstract |
| 1. Assessment of the efficacy of acupuncture and chiropractic on treating Cervical spondylosis radiculopathy: A systematic review and meta-analysis | Conference abstract |
| 1. Effectiveness of acupuncture for treatment of diabetic peripheral neuropathy | Conference abstract |
| 1. Effectiveness of rehabilitation training combined acupuncture for the treatment of neurogenic bladder secondary to spinal cord injury | Conference abstract |
| 1. Performing a large number of overlapping meta-analyses assessing acupuncture efficacy for smoking cessation to explore vibration of effects | Conference abstract |
