# Supplementary material for: Low methodological quality of systematic reviews on acupuncture: a cross-sectional study
Source: BMC Med Res Methodol. 2021 Oct 30;21:237. doi: 10.1186/s12874-021-01437-0 (PMC8557536; doi:10.1186/s12874-021-01437-0)
Supplement: Supplementary file 3 — Additional file 3: STROBE Statement—Checklist of items that should be included in reports of cross-sectional studies. [file 12874_2021_1437_MOESM3_ESM.docx]

STROBE Statement—Checklist of items that should be included in reports of ***cross-sectional studies***

|  | Item No | Recommendation | Page No |
| --- | --- | --- | --- |
| **Title and abstract** | 1 | (*a*) Indicate the study’s design with a commonly used term in the title or the abstract | 1 |
|  |  | (*b*) Provide in the abstract an informative and balanced summary of what was done and what was found | 2 |
| Introduction | | | |
| Background/rationale | 2 | Explain the scientific background and rationale for the investigation being reported | 4 |
| Objectives | 3 | State specific objectives, including any prespecified hypotheses | 4 |
| Methods | | | |
| Study design | 4 | Present key elements of study design early in the paper | 5 (under the heading of “Literature search”) |
| Setting | 5 | Describe the setting, locations, and relevant dates, including periods of recruitment, exposure, follow-up, and data collection | 5 (under the headings of “Eligibility criteria” and “Literature search”) |
| Participants | 6 | (*a*) Give the eligibility criteria, and the sources and methods of selection of participants | 5 (under the heading of “Eligibility criteria”) |
| Variables | 7 | Clearly define all outcomes, exposures, predictors, potential confounders, and effect modifiers. Give diagnostic criteria, if applicable | 6 (under the heading of “Literature screening and data extraction”)  7 (under the heading of “Methodological quality assessment”) |
| Data sources/ measurement | 8* | For each variable of interest, give sources of data and details of methods of assessment (measurement). Describe comparability of assessment methods if there is more than one group | 7 (under the heading of “Methodological quality assessment”) |
| Bias | 9 | Describe any efforts to address potential sources of bias | 6 (under the heading of “Literature screening and data extraction”)  7 (under the heading of “Methodological quality assessment”) |
| Study size | 10 | Explain how the study size was arrived at | 5 (under the heading of “Literature search”) |
| Quantitative variables | 11 | Explain how quantitative variables were handled in the analyses. If applicable, describe which groupings were chosen and why | 8 (under the heading of “Data analysis”) |
| Statistical methods | 12 | (*a*) Describe all statistical methods, including those used to control for confounding | 8 (under the heading of “Data analysis”) |
|  |  | (*b*) Describe any methods used to examine subgroups and interactions | NA |
|  |  | (*c*) Explain how missing data were addressed | NA |
|  |  | (*d*) If applicable, describe analytical methods taking account of sampling strategy | NA |
|  |  | (*e*) Describe any sensitivity analyses | NA |
| Results | | | |
| Participants | 13* | (a) Report numbers of individuals at each stage of study—eg numbers potentially eligible, examined for eligibility, confirmed eligible, included in the study, completing follow-up, and analysed | 8 (under the heading of “Literature selection”) |
|  |  | (b) Give reasons for non-participation at each stage | Figure 1 |
|  |  | (c) Consider use of a flow diagram | Figure 1 |
| Descriptive data | 14* | (a) Give characteristics of study participants (eg demographic, clinical, social) and information on exposures and potential confounders | 9 (under the heading of “Bibliographical characteristics of the included systematic reviews”) |
|  |  | (b) Indicate number of participants with missing data for each variable of interest | NA |
| Outcome data | 15* | Report numbers of outcome events or summary measures | 9 (under the heading of “Bibliographical characteristics of the included systematic reviews”) |
| Main results | 16 | (*a*) Give unadjusted estimates and, if applicable, confounder-adjusted estimates and their precision (eg, 95% confidence interval). Make clear which confounders were adjusted for and why they were included | 9 (under the headings of “Methodological quality” and “Relationship between bibliographical characteristics and overall methodological quality”) |
|  |  | (*b*) Report category boundaries when continuous variables were categorized | NA |
|  |  | (*c*) If relevant, consider translating estimates of relative risk into absolute risk for a meaningful time period | NA |
| Other analyses | 17 | Report other analyses done—eg analyses of subgroups and interactions, and sensitivity analyses | NA |
| Discussion | | | |
| Key results | 18 | Summarise key results with reference to study objectives | 11 (under the heading of “Summary of results”) |
| Limitations | 19 | Discuss limitations of the study, taking into account sources of potential bias or imprecision. Discuss both direction and magnitude of any potential bias | 14 (under the heading of “Strengths and limitations”) |
| Interpretation | 20 | Give a cautious overall interpretation of results considering objectives, limitations, multiplicity of analyses, results from similar studies, and other relevant evidence | 12 (under the heading of “Comparison with other cross-sectional studies on systematic review rigour”)  13 (under the heading of “Recommendation for future systematic reviews”)  15 (under the heading of “Implications”) |
| Generalisability | 21 | Discuss the generalisability (external validity) of the study results | NA |
| Other information | | | |
| Funding | 22 | Give the source of funding and the role of the funders for the present study and, if applicable, for the original study on which the present article is based | 18 |

*Give information separately for exposed and unexposed groups.

**Note:** An Explanation and Elaboration article discusses each checklist item and gives methodological background and published examples of transparent reporting. The STROBE checklist is best used in conjunction with this article (freely available on the Web sites of PLoS Medicine at http://www.plosmedicine.org/, Annals of Internal Medicine at http://www.annals.org/, and Epidemiology at http://www.epidem.com/). Information on the STROBE Initiative is available at www.strobe-statement.org.
